# The Impact of Climate Change on Cancer Surgery and Healthcare Delivery: A Review of Environmental and Surgical Challenges

**DOI:** 10.1007/s10552-025-01999-0

**Published:** 2025-04-22

**Authors:** Shaneeta Johnson, Timia’ Sims, Evangeline Obichere, Jacqueline McWhorter, Jennifer Edwards, Ashley Lewis, Hadiyah-Nicole Green

**Affiliations:** 1 Department of Surgery, Morehouse School of Medicine, Atlanta, Georgia; 2 Morehouse School of Medicine, Satcher Health Leadership Institute, Atlanta, Georgia; 3 Ora Lee Smith Cancer Research Foundation, Atlanta, Georgia; 4 Renewell Foundation, Dallas, TX USA; 5 Atlanta Veterans Affairs Hospital, Atlanta, Georgia

**Keywords:** Climate change, Cancer surgery, Healthcare delivery, Air pollution, Environmental justice

## Abstract

**Purpose:**

More than 10 million annual global cancer deaths are exacerbated by the impact of climate change and environmental determinants of health. This brief report provides a summary of and mitigating recommendations for the complex intersection between climate change and surgical cancer care.

**Methods:**

A review of scientific literature from the last 10 years was conducted to assess the current impact of climate change on cancer care with a focus on surgical interventions. Studies with an impact score of 6 or higher and the keywords of climate change, extreme weather, cancer care, and surgery were reviewed. After removing duplicates and excluded studies, 30 studies remained and were reviewed by two reviewers.

**Results:**

Climate-related factors impacting surgical care result in a myriad of healthcare impacts, including disruption of services, impact on patient outcomes and survival, as well as an overburdening of hospital and surgical services.

**Conclusion:**

Climate change, including extreme weather events, threatens cancer surgical care and delivery by exacerbating comorbidities, disrupting healthcare systems, and increasing disparities in cancer care. Climate change is a burgeoning threat to global health, cancer care, patients, and communities.

## Introduction

Each year cancer causes approximately 10 million deaths globally [1]. This cancer mortality incidence is exacerbated by the evolving threat of climate change on healthcare systems and patient care. It is estimated that greater than 70% to 90% of the most common cancers in humans are attributable to extrinsic factors including environmental pollutants [2]. Environmental impacts, including outdoor and indoor air pollution, extreme heat, and extreme weather such as flooding, wildfires, tornadoes, and hurricanes, significantly impact health outcomes [3, 4]. Air pollution is estimated to have caused premature deaths totaling almost seven million annually, often compounded by the disruption of health services [3]. The World Health Organization (WHO) has recognized climate change as the most significant threat to health, and the impact of environmental factors on physical, economic, mental, and social health is often overlooked [3]. Extreme weather events, worsening air pollution, extreme heat, sea level rise, flooding, and changes in water and food quality and supply all impact the health of cancer patients. These climate change events pose significant challenges for healthcare providers and health systems, including the destruction of infrastructure, supply disruption, and difficulties with access to medications and care, including cancer care [5]. Hurricanes Helene and Milton in the United States (US) brought widespread healthcare impacts in 2024. [6, 7]. The consequences of climate change-related impacts like extreme weather, extreme heat, and air pollution are implicated in delaying the diagnosis, contributing to delayed or decreased access to care and treatment of cancer, and poorer patient outcomes [4]. Surgical intervention remains a cornerstone of the standard of care for many patients presenting with a cancer diagnosis. The relationship between climate change, cancer care, cancer surgery, and cancer outcomes is underexplored but increasingly relevant as climate change accelerates and disrupts the safe and timely delivery of cancer care.

This report explores the deleterious impacts of the complex intersection between climate change and cancer surgical care. It assesses the relationship between climate-related factors, specifically air pollution and extreme weather events, and how they disrupt surgical care delivery and impact patient outcomes and healthcare systems. We offer a focused narrative exploring mitigation and adaptation strategies to lessen the future impact of climate change on cancer surgical care.

## Air pollution, wildfires, and oncologic risk

Globally, air pollution is one of the biggest health threats, and long-term exposure to air pollution increases the global burden of disease [8, 9]. According to WHO, air pollution is responsible for approximately 7 million premature deaths globally every year [3]. Particulate matter (PM) refers to fine particles or liquid particles that have been suspended in the air, typically grouped into three categories: particles with diameters of 10 um or less (PM10), fine particles with diameters of 2.5 um or less (PM2.5), and ultrafine particles with diameters less than 0.1 μm. Increased levels of atmospheric pollution attributed to wildfires from climate change, indoor sources of air pollution, and an increase in temperatures can account for increased cancer mortality risk, including lung, breast, and other cancers [10, 11]. In addition, greenhouse gas emissions, deforestation, and industrial processes, also contribute to the continuous increase in air pollution and are projected to intensify [12]. These rising and ubiquitous pollutants often exceed the health-based air-quality guidelines revised in 2021 by WHO, at 5 μg/m^3^ for an annual average concentration of PM2.5 [10, 11].

In 1970, the United States passed the Clean Air Act, which regulates air emissions to protect public health and the environment (12 μg/m^3^) [10, 13]. In 2024, the U. S. Environmental Protection Agency released its new standards for particulate matter, lowering the national ambient air quality standards to 9 μg/m^3^ [13]. Human and experimental model studies support the linkage of particulate matter from ambient air pollution to lung cancer, estimated to account for hundreds of thousands of deaths annually [4, 11]. Persistent exposure to ambient pollution is associated with chronic inflammation in lung tissue, oxidative stress, tissue damage, and genetic instability [10, 11]. Although the Clean Air Act has contributed to the decrease of key pollutants, the challenge remains to effectively address climate change and emissions from existing and new sources and sectors [10].

Wildfires release carcinogens and significantly contribute to air pollution, including being a substantial source of PM [10, 14, 15]. Hazardous carcinogens such as polycyclic aromatic hydrocarbons (PAHs), benzene, heavy metals, and formaldehyde are released from wildfires [15]. Cancer patients, particularly those with weakened immune systems and respiratory symptoms, face elevated health risks, aggravation, and potential disruptions to their treatment plans when exposed to these toxins [14]. This exposure is also associated with an increased risk of respiratory and cardiovascular problems [10]. Exposure can raise the likelihood of complications during surgery, anesthesia, and recovery, often linked to increased frailty among cancer patients due to exposure [10, 14]. For example, exposure to wildfire toxins during the first year after lung cancer surgery has been associated with worse survival outcomes [14].

Early detection and interventions have been shown to save the lives of cancer patients [1, 5]. However, hospital closures or limited operational hours resulting from wildfires can cause delays in scheduling and delivering time-sensitive cancer treatments, including surgery, radiation therapy, and chemotherapy. In California, wildfire events disrupted the continuity of cancer care, resulting in missed radiotherapy visits [16]. These delays in diagnosing and treating cancer due to extreme weather events can result in patients presenting with more advanced tumors, worsened prognosis, and increased mortality [14].

A recent study of over 400,000 non-small cell lung cancer (Stage I–III) patients undergoing resection demonstrated that those who were exposed to wildfires within one year of discharge had worse survival rates than those who were not exposed. Time intervals examined included exposure within three months, 4–6 months, and 7–12 months [14]. The study findings reveal that poor air quality due to wildfires can affect lung cancer patients’ recovery and survival. [14, 16]. Cancer patients often remain in environments with the same pollutants after receiving care, which may adversely impact their recovery, compromise the success of their treatment, and lead to additional health complications [14].

Climate change has substantially increased the frequency and intensity of wildfires, creating significant challenges for consistency and continuity of oncological care [10, 14, 16]. Additionally, air pollution levels and smoke exposure have made cancer patients in remission vulnerable to long-term cardiovascular and respiratory damage, posing significant challenges [10].

## Impact of climate change on surgical care delivery and perioperative risk

### Perioperative frailty

Particulate matter 2.5, based on air quality average concentration guidelines, has been shown to worsen cardiopulmonary function which can increase the risk of complications intraoperatively and postoperatively, making anesthesia and recovery challenging [8, 10]. Air pollution negatively impacts cancer patients and cancer surgery from exposure to recovery, and exposure prior to surgery can cause damage to blood vessels and inflammation due to PM [10]. During surgery, anesthesia delivery may also be impacted by existing cardiovascular system damage, systemic inflammation, and compromised blood vessels. Additionally, cancer patients’ exposure to air pollution can exacerbate their recovery conditions [14]. Challenges presented include respiratory and cardiovascular issues that affect the delivery of anesthesia, in turn increasing the risk of surgical complications [8]. Postoperative complications during recovery may also occur as exposure to pollutants can lead to increased susceptibility to viral infections in the respiratory system, resulting in inflammation and slowed recovery [10]. Addressing these risks specifically for cancer patients can enhance surgical outcomes and recovery.

### Extreme weather impacts on health systems and healthcare delivery

Oncologic surgical care requires timely delivery of services that may be significantly interrupted and impacted by extreme weather events [4, 17]. Furthermore, early detection and early interventions have been shown to save the lives of cancer patients [4]. Climate change and associated extreme weather events increasingly compromise healthcare systems, services, and patient outcomes [3, 7]. When evacuations and facility closures occur, cancer patients often face interruptions in their care, losing access to essential oncology resources, which can lead to worse health outcomes. These challenges further complicate cancer care management, making it harder for patients to maintain a consistent treatment plan [15, 16]. Disruptions in care may include loss of power and damage to electronic medical record systems due to flooding, erosion, and other infrastructure damage, further interrupting patient care [17, 18].

Currently, nearly 3.6 billion individuals live in areas that are highly vulnerable to the continual effects of climate change [3]. For instance, hurricanes Helene and Milton struck the southeastern U.S. in September and October 2024, significantly impacting surgical care, infrastructure of roads, hospitals, clinics, and the supply chain for vital products [6, 7]. These storms resulted in significant morbidity and mortality from both direct and indirect effects. Intravenous fluid shortages hindered the delivery of essential services ranging from dialysis to elective surgery. Evacuation of hospital staff and patients from impacted and compromised health system buildings also impacted healthcare delivery in these affected communities. Hurricanes Harvey, Sandy, and Katrina caused widespread disruption along the East and Gulf Coasts, damaging food sources, city infrastructure, and property [19]. Research following Hurricane Sandy revealed disparities in the storm’s impact based on socio-demographic factors [19]. Studies utilizing FEMA and self-assessed measurements support the findings that individuals living in impoverished areas and older individuals are most likely to experience the effects of flooding [19]. However, those living in areas with higher household incomes are least likely to experience the effects of flooding [19]. Hurricane Katrina impacted access to oncology care for years and was associated with a lower breast cancer survival rate [4]. Extreme weather events like flooding, which can be a part of the aftermath of hurricanes, negatively influence the continuity of care for patients [5]. However, patients with socioeconomic limitations and other marginalized populations may have increased difficulty preparing and responding to climate change effects as well as continuing their cancer treatments [4, 18, 19].

Extreme weather and other climate change impacts disrupt cancer care delivery [17, 18]. Impacted services and delays of vital care, such as chemotherapy treatments and scheduled oncologic surgeries, may put patients at risk for cancer progression and increased mortality rates [1, 18]. Major disasters place a heavy strain on elective and emergency health care, often disrupting the continuum of care needed by cancer patients [7, 18]. These delays include loss of power and disruption of the electronic medical record system due to flooding, erosion, and other infrastructure damage, further interrupting patient care [17, 18]. One study noted that 23.9% of National Cancer Institute (NCI)-)-designated cancer centers provided general emergency preparedness information, and only seven NCI centers provided cancer-specific emergency preparedness information regarding climate-related disasters [20]. Research supports that patients currently undergoing radiation treatments have worse overall survival rates after a hurricane, likely due to multifactorial etiologies, including treatments not being prioritized and an overall delay in their care [20, 21]. After disaster strikes, pertinent medications and treatment specialists are more difficult to locate, which can disrupt treatment paradigms and increase anxiety and depression for cancer patients [9, 17]. Patients with cancer have a higher incidence of depression and anxiety; however, studies demonstrate that the mental health impacts of extreme disaster events can persist for up to 15 years, further exacerbating mental health strain [6, 17, 19].

## Solutions and recommendations

Addressing the impact of climate change on the timeliness and quality of cancer care delivery requires the elimination of gaps in our practices and strategies to mitigate risks and ensure climate-resilient healthcare infrastructure and systems. To address these urgent issues, we propose the following recommendations (Table 1.) to help mitigate the effects of climate change, particularly for cancer patients in surgical environments. Moreover, these strategies specifically aim to address the environmental health disparities that lead to increased incidence of adverse health outcomes and cancer incidence in marginalized, frontline, and fenceline communities.

1. *Create tools to evaluate patient risk from climate change.* There is currently a shortage of patient and provider knowledge regarding the risk profile of patients presenting for surgical cancer care. Creating a standard technique to measure climate change-related risk preoperatively can help identify patients who may be more vulnerable to complications. The National Oceanic and Atmospheric Administration (NOAA), Environmental Protection Agency (EPA), and Center for Disease Control and Prevention (CDC) have developed multiple climate risk tools; however, these tools should be integrated into the healthcare workflow to better prevent, predict, and respond to climate-associated health risks [22].
2. *Reduce Air Pollution Exposure.* Reducing exposure to air pollution from wildfires and outdoor and indoor pollution sources will improve the health risks and perioperative profile of oncologic surgical patients [14, 16]. Healthcare facilities should focus on purifying air and ensuring proper ventilation. It is essential to monitor air quality in real-time and educate patients about their individual risks associated with air pollution [9, 11].
3. *Increase the Use of Home Health and Telehealth.* Sustainable care models should include telehealth and home-based treatment options as clinically appropriate. These can reduce the need for patient travel, particularly during emergencies, and minimize the carbon footprint associated with traditional in-person care settings and transportation [3, 7, 17, 20, 23, 24].
4. *Adopt renewable energy and sustainable surgical practices.* The healthcare system is a significant contributor to greenhouse gas emissions [24, 25]. It is imperative to reduce reliance on fossil fuels and adopt policies to reduce carbon footprints (i.e., the Health and Human Services (HHS) pledge and the Joint Commission on Accreditation of Healthcare Organization’s (JCAHO) voluntary standards) [18]. Systems should also implement energy-saving measures like occupancy-based ventilation and HVAC setbacks. The operating room consumes a significant amount of energy, and implementing strategies such as light-emitting diode (LED) lighting or solar energy can significantly decrease the carbon footprint. Reducing the use of single-use items and encouraging reusable instrument use will also lower waste.[18, 23].
5. *Resilient Healthcare Infrastructure.* Healthcare facilities, particularly in low-income communities, may lack climate-resilient healthcare infrastructure, and available facilities are typically outdated, underfunded, and understaffed, resulting in long wait times and poorer health outcomes. Efforts and funding should be invested in healthcare infrastructure to improve facilities’ resilience to flooding, strong winds, extreme weather, and other climate change impacts. [4, 5, 7, 22, 23, 26].
6. *Emergency preparedness plan*. Acute injuries are the primary cause of death immediately after a disaster [22, 26]. Health systems must have disaster preparedness plans with contingencies for extreme weather events to ensure continuity of and improved cancer care outcomes, including reducing critical injuries and deaths during emergencies [19, 20]. Additionally, healthcare facilities should conduct annual emergency preparedness drills to train healthcare staff on how to respond to disasters safely and [17, 26, 27].
7. *Equitable Resource Distribution to Marginalized Communities.* Low-income, racially ethnic, and rural communities are disproportionately affected by climate change and often have inequitable resource allocation [4, 5, 12, 19, 22, 25, 28]. A long-standing history of structural and systemic barriers, such as redlining, fewer healthcare facilities in low-income communities, and underserved health professional shortage areas, directly impacts health outcomes for cancer patients in these areas. To reduce the healthcare disparity gap, it is essential to identify at-risk communities and develop funding models that prioritize these communities for adequate screening, treatment, care, and emergency planning. [18, 19, 22, 24].
8. *Increase education about climate change and its impact on point of care.* Many individuals, including healthcare professionals, may not fully understand the effects of climate change, and some may be resistant to altering their lifestyle or everyday habits [25, 29]. Climate change education should be incorporated into healthcare professional curriculums. Patient and community engagement campaigns are needed to create an awareness of the growing impact of climate change on oncologic risk and cancer surgical care. It is essential to adopt best practices globally to ensure the incorporation of solutions and diverse scientific knowledge [18, 23, 25].
9. *Increase community funding.* Federal, state, and local funding should be invested in healthcare infrastructure and education to improve health facilities and staff recruitment. These funds can enhance community resilience against flooding, strong winds, extreme weather, and other climate change impacts. Investments in the infrastructure can also intersect with funding emergency preparedness, eco-friendly planning, risk tools, telehealth equipment, and educational resources [3, 5, 7, 17, 22].

**Table 1:** Potential Solutions for Mitigating the Impact of Climate Change On Surgical Cancer Care

Potential solutions for mitigating impact	Description	Benefits
1. Creating and adopting a standard and reliable risk assessment tool [22]	Develop and integrate appropriate risk tools in healthcare to assess climate-related health risks in cancer patients e.g. the EPAs Climate Mapping for Resilience and Adaptation (CMRA) tool, the CDCs Health Tracker, and NOAAs Climate Resilience Toolkit	Better identification and stratification of cancer patients with a higher risk of negative outcomes due to climate change
2. Reducing air pollution exposure [9, 11, 14, 16]	Implement air quality enhancements such as the use of air filtration and conditioning systems, reducing the use of gas stoves, and monitoring of air quality in real-time	Reduced exposure to air pollutants/carcinogens, lower respiratory and surgical complications
3. Home health and telehealth use [3, 7, 17, 20, 23, 24]	Expand access to home health and telehealth services to maintain high-quality cancer care during emergencies	Reduced cancer care disruption and improved disease prognosis
4. Eco-friendly practices [18, 23–25]	Shift to renewable energy, public transportation, using less plastic, reducing waste in the environment and operating rooms	Reduced greenhouse gas emissions, pollution, carbon footprint, and energy costs
5. Reinforcement of healthcare infrastructure [4, 5, 7, 22, 23, 26]	Build and upgrade facilities that are resistant to extreme weather events	Minimized disaster impact on infrastructure and reduced disruption to continuity of care
6. Emergency preparedness [17, 19, 20, 22, 26, 27]	Establish safety protocols and evacuation plans that consider cancer patients in preparation for projected natural disasters	Improved cancer patient and staff safety, reduced gaps in care
7. Resource distribution to marginalized communities [4, 5, 12, 18, 19, 22, 24, 25, 28]	Identify the most vulnerable communities and develop funding models that prioritize these communities. Increase access to community resilience hubs	Closed healthcare disparity gap, Reduced cancer risk from exposure to carcinogens
8. Community education and [18, 23, 25, 29]	Develop programs to educate the community about climate change and its effects on cancer patients	Increased understanding of the community, cancer patients, and caregivers to take informed actions and change daily habits
9. Funding allocation [3, 5, 7, 17, 22]	Advocate for increased investment in health system infrastructure focusing on upgrades, expansions, and community investments	Increased community and health system resilience and access to high-quality cancer care

Promising strategies to address the impact of climate change on oncologic surgical care encompass a range of interventions. These include individual-level interventions to reduce personal cancer risks and systemic strategies that health systems can adopt to increase their resilience and adaptation. Opportunities exist to mitigate inequities in infrastructure, planning, and resource allocation. Overall, efforts to reduce exposure, improve home health delivery, modify surgical practices, improve infrastructure, proactively plan, distribute resources equitably, and educate the community can enhance the preparation and management of climate change’s effects on oncologic surgical care [17, 23, 24].

Successfully implementing these strategies requires coordination from policymakers, communities, and industries. Healthcare systems will be better able to respond to the evolving threats posed by climate change while providing optimal care for cancer patients by successfully implementing resilience and adaptation strategies [25, 26].

## Conclusion

**Fig. 1:**
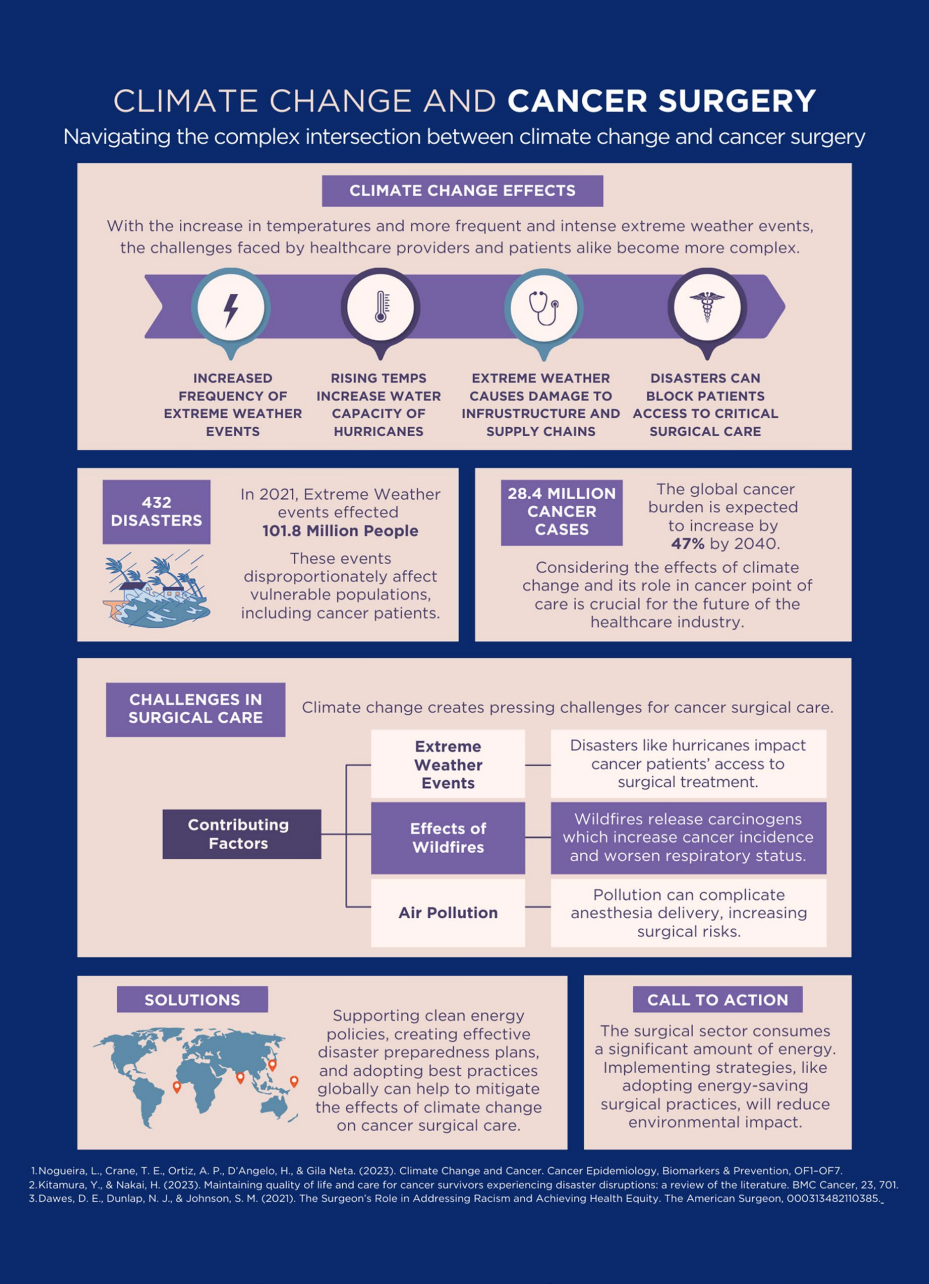
Climate Change and Cancer Surgery [17, 18, 28]

Cancer patient outcomes and survival are multifactorial. However, to enhance patient outcomes, it is vital to increase awareness of the ongoing health implications of climate change within surgical oncology (Fig. 1). Implementing multidisciplinary approaches to mitigation and adaptation with the development of relevant contingency plans for surgical procedures during extreme weather events and other climate change impacts is essential for maintaining the safety of surgical cancer care treatment [18].

Previous research has outlined strategies that can help reduce the carbon footprint and improve surgical outcomes for cancer patients. However, there are several barriers to effectively implementing these decarbonization strategies. These challenges include resources and funding availability, policy and industrial pushbacks, and a reluctance of the population to change established practices [18].

Examining inequities in the experiences of cancer patients facing climate change in rural and underserved communities can provide valuable insights into emergency preparedness [4, 5, 22, 24]. By addressing these inequities, we can ensure that all patients have appropriate and equitable access to cancer care, treatment, and resources during disasters. Further research is needed to identify best practices for patient-centered climate change emergency preparedness [17, 18].

## Data Availability

No datasets were generated or analysed during the current study.
